# On-Orbit Absolute Radiometric Calibration and Validation of ZY3-02 Satellite Multispectral Sensor

**DOI:** 10.3390/s22052066

**Published:** 2022-03-07

**Authors:** Hongzhao Tang, Junfeng Xie, Xinming Tang, Wei Chen, Qi Li

**Affiliations:** 1 School of Earth and Space Sciences, Peking University, Beijing 100871, China; liqi@pku.edu.cn; 2 Land Satellite Remote Sensing Application Center, Ministry of Natural Resources, Beijing 100094, China; xiejf@lasac.cn (J.X.); tangxm@lasac.cn (X.T.); 3 College of Geoscience and Surveying Engineering, China University of Mining and Technology, Beijing 100083, China; chenw@cumtb.edu.cn

**Keywords:** radiometric calibration, TOA radiance, reflectance-based approach, the Baotou site, ZY3-02 satellite

## Abstract

This study described the on-orbit vicarious radiometric calibration of Chinese civilian high-resolution stereo mapping satellite ZY3-02 multispectral imager (MUX). The calibration was based on gray-scale permanent artificial targets, and multiple radiometric calibration tarpaulins (tarps) using a reflectance-based approach between July and September 2016 at Baotou calibration site in China was described. The calibration results reveal a good linear relationship between DN and TOA radiances of ZY3-02 MUX. The uncertainty of this radiometric calibration was 4.33%, indicating that radiometric coefficients of ZY3-02 MUX are reliable. A detailed discussion on the validation analysis of the comparison results between the different radiometric calibration coefficients is presented in this paper. To further validate the reliability of the three coefficients, the calibrated ZY3-02 MUX was compared with Landsat-8 Operational Land Imager (OLI). The results also indicate that radiometric characteristics of ZY3-02 MUX imagery are reliable and highly accurate for quantitative applications.

## 1. Introduction

The Ziyuan-3 (ZY3) satellite constellation is China’s civilian high-resolution stereo mapping satellite system, consisting of four satellites: ZY3-01, ZY3-02, ZY3-03, and ZY3-04. The ZY3-01 satellite was launched into orbit on 14 January 2012, is the first operational satellite of the ZY3 series, and completed its official mission and stopped operation in 2017. As its continuation, ZY3-02 was successfully launched at Taiyuan Satellite Launch Center on 30 May 2016, and is still in operation. ZY3-02 carried a multispectral imager (MUX) and three panchromatic cameras (TLC). The multispectral imagery provides repetitive acquisition of multispectral data with a high resolution of 5.8 m, while panchromatic imagery of 2.1-m resolution is mainly used for 1:50,000 scale topographic mapping. The primary purpose of the ZY3 satellite constellation is to satisfy China’s need for satellite imagery for surveying and mapping and the geographic information system industry. Most studies of the ZY3-01 and ZY3-02 satellites have focused on geometric calibration, and minimal work has been performed on the radiometric calibration and validation, while quantitative applications such as surface albedo retrieval and aerosol parameter retrievals of ZY-01 and ZY-03 images require reliable radiometric information. Since the launch of ZY3-02, ZY3 satellite products have successfully played an important role in China’s environmental, agricultural, and other quantitative applications, which require reliable radiometric information.

The on-orbit absolute radiometric calibration of the satellite sensor is a critical activity in providing highly accurate quantitative measurements of the Earth’s surface [1]. Although the technical parameters of the satellite sensor have been accurately measured in the laboratory before launch, data from satellites are sensitive to post-launch changes, regardless of the quality of the calibration data. The absolute radiometric calibration of a satellite sensor is essential for maintaining stable satellite data quality and for identifying the relationship between the image digital number (DN) and at-sensor radiance [2,3]. Reflectance-based vicarious calibration was recognized as one of the most reliable approaches for on-orbit absolute radiometric calibration of satellite sensors [4]. This approach has been employed for on-orbit absolute radiometric calibration of numerous satellite sensors, such as Moderate Resolution Imaging Spectroradiometer (MODIS), Landsat-7 Enhanced Thematic Mapper Plus (ETM+), Landsat-8 Operational Land Imager (OLI), and the sensors loaded on IKONOS and SPOT satellites, as well as the multispectral cameras on board China’s Fengyun (FY), Ziyuan (CBERS), and Huanjing (HJ) satellites [5,6,7,8,9,10,11,12].

In this paper, we describe the methods and results of a reflectance-based vicarious calibration campaign that was conducted between July and September 2016 at Baotou Site, located in Inner Mongolia, China. A series of gray-scale permanent artificial targets and infrastructure has been built in the Baotou site to provide effective support for satellite sensor on-orbit calibration. A set of several radiometric calibration tarpaulins with nominal reflectance of 5%, 20%, 40%, and 60% were designed for the on-orbit absolute radiometric evaluation of the ZY3-02 satellite multispectral imager. The reflectance-based vicarious calibration approach relies on the synchronous measurement of the surface spectral reflectance and atmospheric parameters. In this campaign, the synchronous measurements of surface and atmospheric conditions (including aerosol optical depth, amount of water vapor, aerosol inversion products) at the Baotou site at the time of ZY3-02 satellite overpass. The synchronous measurement of the surface spectral reflectance and atmospheric parameters is coupled with exoatmospheric solar irradiance spectrum and relative spectral response of the sensor as inputs into a radiative transfer model to compute the at-sensor spectral radiance. The relationship between the at-sensor radiance and the DN recorded in the ZY3-02 satellite image is identified. We can obtain several different radiometric calibration coefficients determined with different target regions, such as gray-scale permanent artificial targets or radiometric calibration tarps with nominal reflectance. A detailed discussion on the validation analysis of the comparison results between the different radiometric calibration coefficients is presented in this paper. The aim of our study is to obtain a reliable and high-accuracy radiometric calibration coefficient for the ZY3-02 satellite sensor.

## 2. Materials

### 2.1. Overview of ZY3-02 Satellite

The ZY3-02 satellite was launched into a sun-synchronous orbit on 30 May 2016. It covers the global region every 59 days. The ZY3 satellite carries a multispectral imager (MUX) and three-line panchromatic cameras (TLC). The multispectral imagery provides repetitive acquisition of multispectral data with a high resolution of 5.8 m at ground sample distance (GSD), while the panchromatic imagery of 2.1 m resolution at GSD is mainly used for topographic purposes and is not discussed in this paper (Table 1).

### 2.2. ZY3-02 Multispectral Imager (MUX)

**Figure 1:**
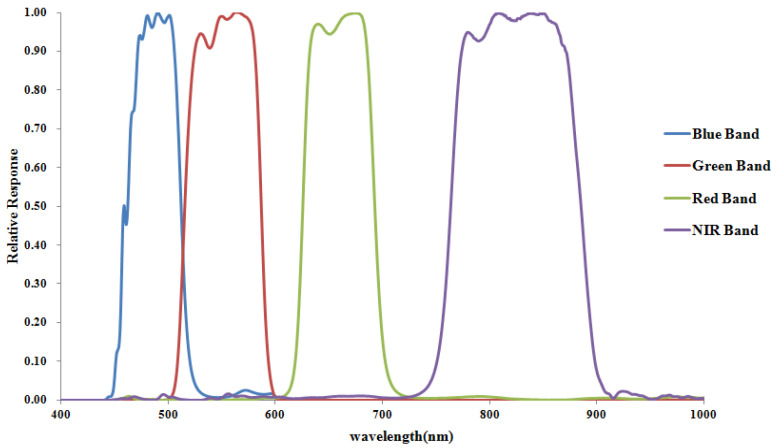
Normalized spectral response function of the ZY3 MUX.

The ZY3-02 multispectral imager produces a GSD equal to 5.8 m at nadir in all four multispectral bands and a swath width of 51 km from a nominal altitude of 506 km. The normalized spectral response function of ZY3-02 MUX is shown in Figure 1. Several of the key characteristics of ZY3-02 MUX are listed in Table 2.

### 2.3. Calibration Site and Targets

#### 2.3.1. Baotou Calibration Site

**Figure 2:**
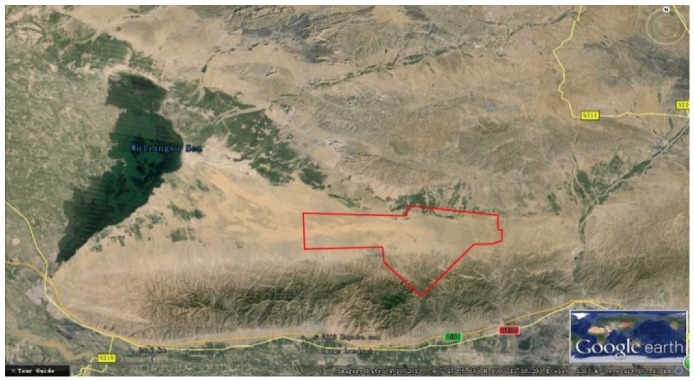
The RadCalNet Baotou site.

In 2013, the Committee on Earth Observation Satellites (CEOS) Working Group on Calibration and Validation (WGCV) Infrared and Visible Optical Sensors Subgroup (IVOS) established the Radiometric Calibration Network (RadCalNet), consisting of four international test sites located in the continental United States, France, China, and Namibia, providing automated in situ measurements and estimates of propagated top of atmosphere (TOA) reflectance [13]. The RadCalNet Baotou site (BTCN) is located in Inner Mongolia, China, about 50 km from Baotou city, as shown in Figure 2 (in red circle). It covers a flat area of approximately 300 km^2^. The site is dominated by sand and bare soil. A series of targets and infrastructure has been built in the Baotou site to provide effective support for sensor aerial test flights, satellite sensor on-orbit calibration and performance evaluation, and remote-sensing product validation [14,15].

#### 2.3.2. The Gray-Scale Permanent Artificial Targets

**Figure 3:**
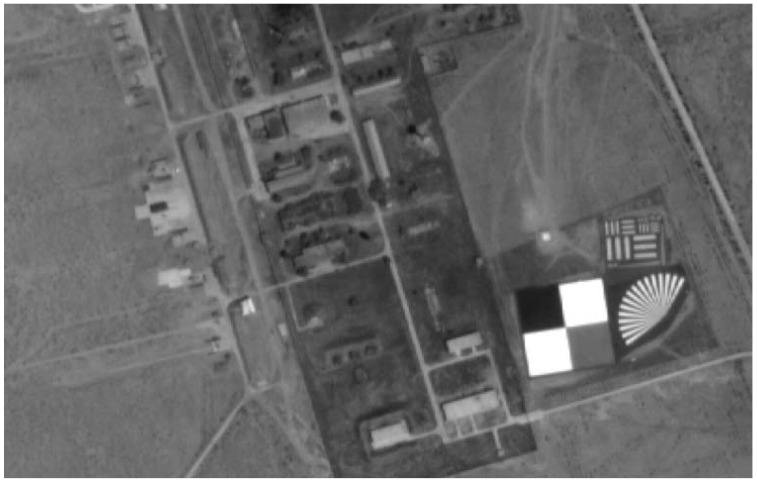
The gray-scale permanent artificial targets at RadCalNet Baotou site.

The Baotou site is currently maintained and operated by the Aerospace Information Research Institute, Chinese Academy of Science. A series of targets and infrastructure has been built in the Baotou site to provide effective support for satellite sensor on-orbit calibration and performance evaluation, and remote-sensing product validation. Figure 3 shows the gray-scale permanent artificial targets. The gray-scale permanent artificial targets are composed of two white, one gray, and one black uniform gravel squares, each of which covers an area of 48 m × 48 m, which can be used for radiometric calibration and validation of high-resolution optical sensors. The white, gray, and black targets with known spectral reflectance (56%, 18%, and 7%) have a fairly flat spectral reflectance [16].

#### 2.3.3. The Calibration Tarps

**Figure 4:**
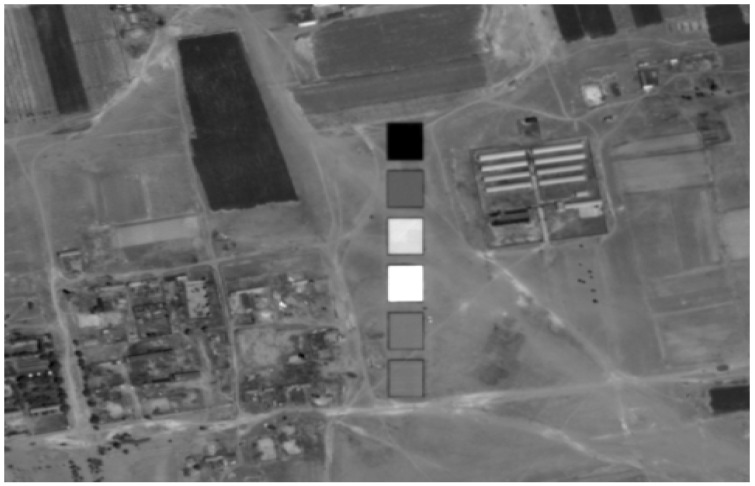
The arrangement of the calibration tarps at the RadCalNet Baotou site.

In order to properly calibrate the ZY3-02 MUX, a set of four radiometric calibration tarps with nominal reflectance of 5%, 20%, 40%, and 60% (with relatively stable reflectance curve around the nominal values with an error of less than 3%) was designed and deployed to the Baotou site. These four radiometric calibration tarps were designed as ideal calibration targets with good Lambertian properties measured and validated at the laboratory, which should be relatively flat with little variation (less than 7% of the nominal values) in the surface feature and be stable in a spectral sense. Two color-scaled radiometric tarps of blue and red were also designed with quasi-Lambertian properties for on-orbit absolute radiometric evaluation. Each of these calibration tarps covers an area of 60 m × 60 m. The spatial variation of each calibration tarp is less than 2%. The laboratory measured bi-directional reflectance factor (BRF) of the calibration tarp shows that the spectral variation of the four radiometric calibration tarps’ reflectance over 400–1000 nm is less than 3%. Figure 4 shows the arrangement of the calibration tarp at the Baotou site during the ZY3-02 calibration campaign.

## 3. Methodology

### 3.1. Radiometric Calibration Approaches

**Figure 5:**
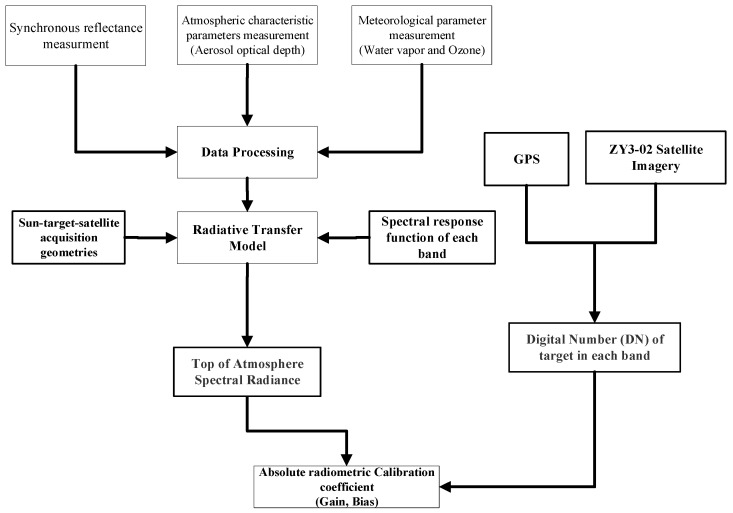
Flow chart of the reflectance-based radiometric calibration approach.

The reflectance-based vicarious calibration approach is the most commonly used method, which relies on synchronous measurement of the surface spectral reflectance and atmospheric parameters (including the aerosol optical depth and amount of water vapor). The measurement of the surface spectral reflectance and atmospheric parameters are required as inputs into a radiative transfer model, such as Moderate Resolution Atmospheric Transmission (MODTRAN 6.0), to compute the TOA spectral radiance. The simulated spectral radiance at the TOA from the radiative transfer model is matched with satellite DN and is used to obtain the sensor’s absolute radiometric calibration coefficients [17,18,19]. The flow chart of the reflectance-based vicarious calibration approach is shown in Figure 5.

### 3.2. Synchronous Measurement of Surface Reflectance

**Figure 6:**
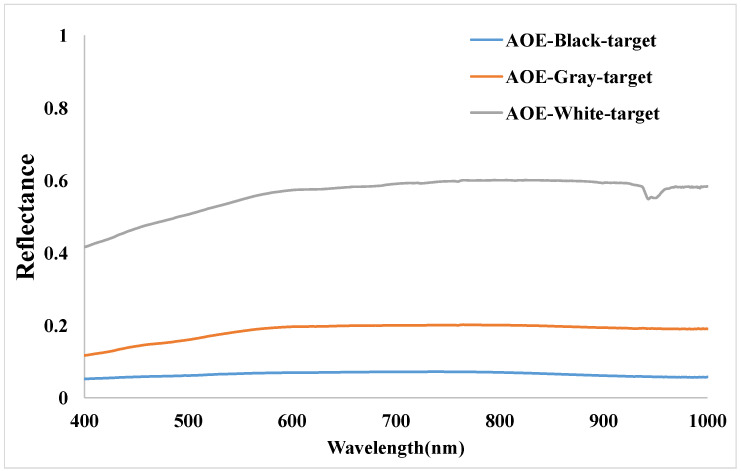
Spectral reflectance measurement data of gray-scale permanent targets.

**Figure 7:**
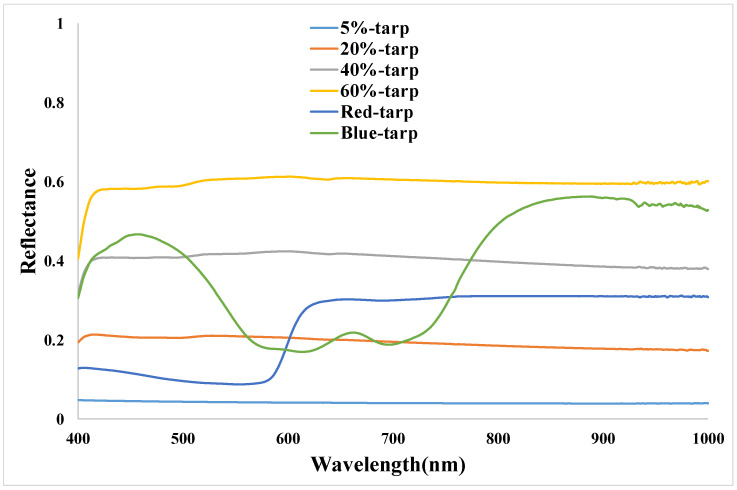
Spectral reflectance measurement data of calibration tarps.

The reflectance measurements of the gray-scale permanent artificial targets and radiometric calibration tarps were collected within 30 min before and after the overpass time of the ZY3-02 satellite. The size of each gray-scale permanent artificial target is 48 m × 48 m, and covers about 8 cross-track pixels and 8 along-track pixels of ZY3-02 imagery. The spectroradiometer was configured to average five spectra per sample, and 16 samples were collected within each single target. During the measurements, the direction of the probe is nearly straight down, and, considering the Lambertian of the target, the error could be neglected [20]. The radiometric calibration tarps were 60 m × 60 m, covering about 10 cross-track pixels and 10 along-track pixels of ZY3-02 MUX imagery, five spectra per sample, and 25 samples were collected within each single tarp. The four gray-scale permanent artificial targets and six calibration tarps gave a total of 214 samples and more than 1000 spectra collected. It took more than 30 min to collect these data. The reflectance was measured by the SVC HR-1024i spectroradiometer (SVC Spectra Vista Corporation), which covered the wavelength range from 350 nm to 2500 nm. SVC HR-1024i is a high-resolution field portable spectroradiometer with a spectral resolution of 1.5 nm in the 350–1000 nm spectral range, 3.8 nm in the 1000–1890 nm spectral range, and 2.5 nm in the 1890–2500 nm spectral range. The output of the SVC software was interpolated at 1 nm intervals in the 350–2500 nm range. During spectra collection, five spectra were collected in each point and these five spectra were averaged to obtain the mean spectrum in this point. Figure 6 and Figure 7 show the reflectance measurements from the targets and tarps in the range of 400–1000 nm.

### 3.3. Synchronous Measurement of Atmospheric Parameters

**Figure 8:**
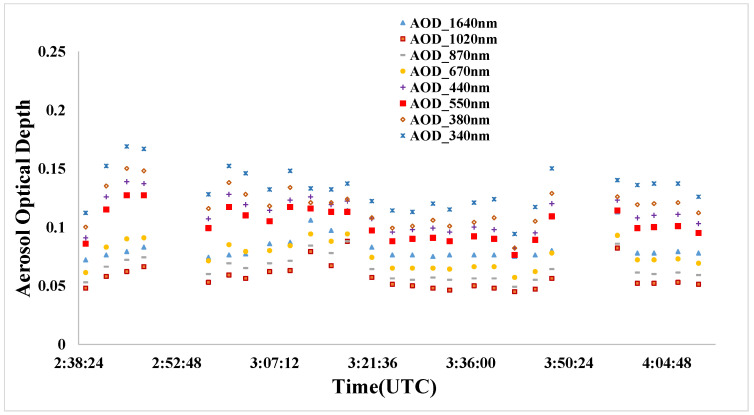
The measurement of atmospheric parameter of AERONET Baotou site.

Aerosol parameters were collected at the time of ZY3-02 satellite overpass. An automatic sun photometer (CIMEL CE318) was used to measure the aerosol optical depth (AOD) and the total columnar water vapor (CWV). The AOD and CWV were retrieved by the AERONET data process center from the CE318 sun photometer observed data at the Baotou site, which joined the Aerosol Robotic Network (AERONET) in September 2013. Figure 8 shows the measurement of atmospheric parameters of the AERONET Baotou site. The AOD at 550 nm is estimated from the Langley algorithm-derived AOD at the 440, 670, 870, and 1020 nm channels with the Ångström empirical equation, as follows:

(1) $$\tau_{\alpha }(\lambda )=\beta \cdot \lambda^{-\alpha }$$

where $\tau_{\alpha }(\lambda )$ is AOD at the wavelength *λ*, *β* is Ångström’s turbidity coefficient, and α is the Ångström exponent; *α* and *β* are independent of the wavelength, and they can be calculated by fitting the AOD at the 440, 670, 870, and 1020 nm channels. Table 3 shows the synchronous measurement of atmospheric parameters at the time of ZY3-02 satellite overpass.

The vertical atmospheric profiles of temperature, humidity, and pressure were collected with a radiosonde balloon at the times of the ZY3-02 satellite overpass. In fact, one of the most important sources of systematic error in the reflectance-based vicarious calibration approach is caused by the assumption of aerosol properties including size distribution, refractive index, etc., which determine the microphysical status of aerosol particles affecting the complex interaction between light and aerosol particles [21]. In remote-sensing applications, the aerosol properties are assumed empirically as several types, resulting in a lot of error in radiometric calibrations. However, AERONET directly produced aerosol properties specifically for this scenario, minimizing the error. The simulation of the radiative transfer model shows that the top of atmosphere spectral radiance is highly sensitive with different aerosol properties in a high-aerosol optical depth condition. In other words, the assumption of aerosol properties would bring a large error in predicting the top of atmosphere spectral radiance [22,23]. In order to reduce the uncertainty caused by the aerosol properties assumption, we used the inversion products of the AERONET Baotou site. AERONET collaboration provides the aerosol inversion products (such as size distribution, refractive index, phase function, and asymmetry factor) of the Baotou site. The aerosol inversion products from AERONET collaboration would be taken as the input of a radiative transfer model to calculate the band-specific TOA radiance in this study, to overcome the abovementioned problems of the reflectance-based approach.

### 3.4. Radiative Transfer Calculations

The Radiative Transfer Model (MODTRAN 6.0) is commonly used for radiometric calibration. The reflectance measurements and the atmospheric parameters are inputs for a Radiative Transfer Model that computes a band-specific TOA spectral radiance (also known as at-sensor radiance). The apparent reflectance of the ZY-3 02 satellite sensor $\rho^{*}(\mu_{s},\phi_{s};\mu_{v},\phi_{v})$ can be expressed as:

(2) $$\rho^{*}(\mu_{s},\mu_{v},\phi_{s}-\phi_{v})=\rho_{a}(\lambda )+\frac{\rho }{1-S(\lambda )\rho }T_{\theta_{s}}(\lambda )T_{\theta_{v}}(\lambda )$$

where $\mu_{s}=\cos\theta_{s}$ and $\mu_{v}=\cos\theta_{v}$ are the cosine values of the solar and viewing zenith angles, $\theta_{s}$ is the solar zenith angle, $\phi_{s}$ is the solar azimuth angle, $\theta_{v}$ is the viewing zenith angle, and $\phi_{v}$ is the viewing azimuth angle. $\rho_{a}(\lambda )$ is the atmospheric path reflectance, S(λ) is the atmospheric spherical albedo, $T_{\theta_{s}}(\lambda )$ is total transmittance from the solar to the earth, and $T_{\theta_{v}}(\lambda )$ is the total transmittance from the earth to the satellite sensor [24]. The band-specific TOA radiance $L(\theta_{s},\phi_{s};\theta_{v},\phi_{v})$ can be determined as follows:

(3) $$L(\theta_{s},\phi_{s};\theta_{v},\phi_{v})=\frac{\mu_{s}E_{s}\rho^{*}}{\pi d^{2}}$$

where $\rho^{*}$ is the apparent reflectance of the satellite sensor, $\mu_{s}=\cos\theta_{s}$ is the cosine values of the solar zenith angles, $\theta_{s}$ is the solar zenith angles, $E_{s}$ is the solar irradiance at the top of atmosphere, and d is the Solar–Earth distance factor.

We acquired one calibratable ZY3-02 MUX image, with a viewing zenith angle of less than 2 degrees under clear-sky conditions. Table 4 shows the geometric conditions of ZY3-02 for radiometric calibration.

## 4. Results

### 4.1. Comparison of Three Radiometric Calibration Results from Different Targets

The radiometric calibration coefficients of ZY3-02 MUX were determined through the least-squares method, linearly fitting band-specific TOA spectral radiance with DNs corresponding to the target regions by using Equation (4):

(4) $$L_{i}=Gain_{i}\times DN_{i}+Bias_{i}$$

where $L_{i}$ is the band-specific TOA spectral radiance with unit $W\cdot m^{-2}\cdot sr^{-1}\cdot \mu m^{-1}$, $DN_{i}$ is the digital number of the satellite imagery in band i, and $Gain_{i}$ and $Bias_{i}$ are the radiometric calibration coefficients *Gain* and *Bias* in band i.

**Figure 9:**
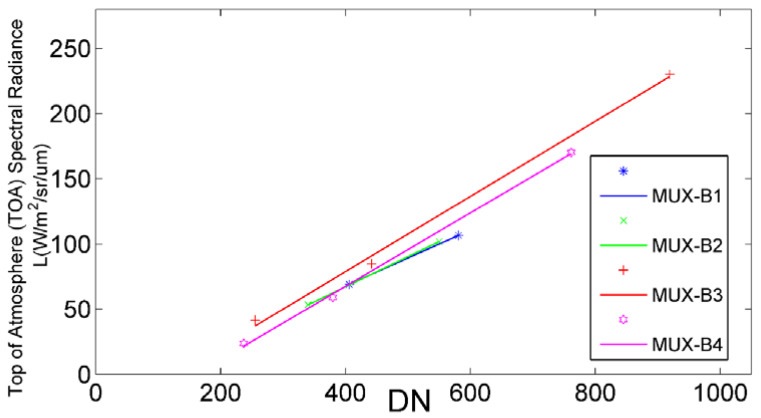
Calibration result of the gray-scale permanent artificial targets (coefficient A).

There would be three radiometric calibration results from different targets. The first radiometric calibration coefficient (coefficient A, as shown in Figure 9) was determined by permanent artificial targets, which just covered three gray-scale reflectances of 7%, 18%, and 56% in 400–1000 nm.

**Figure 10:**
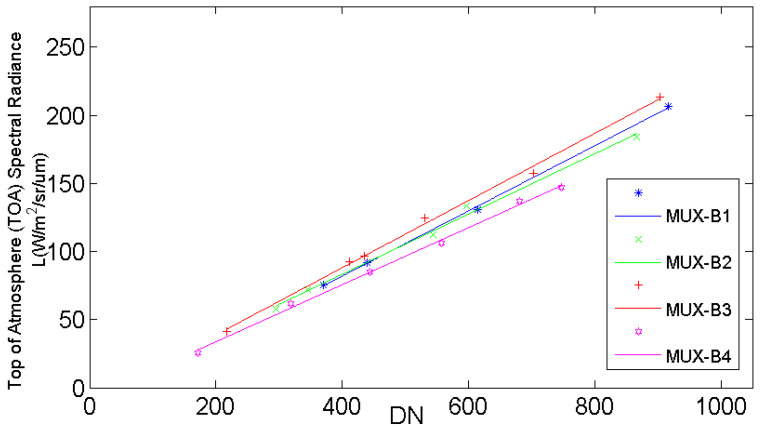
Calibration result of the radiometric calibration tarps (coefficient B).

The second coefficient (coefficient B, as shown in Figure 10) was determined by the four radiometric calibration tarps with four nominal reflectances of 5%, 20%, 40%, and 60%, and two color-scaled radiometric tarps of blue and red.

**Figure 11:**
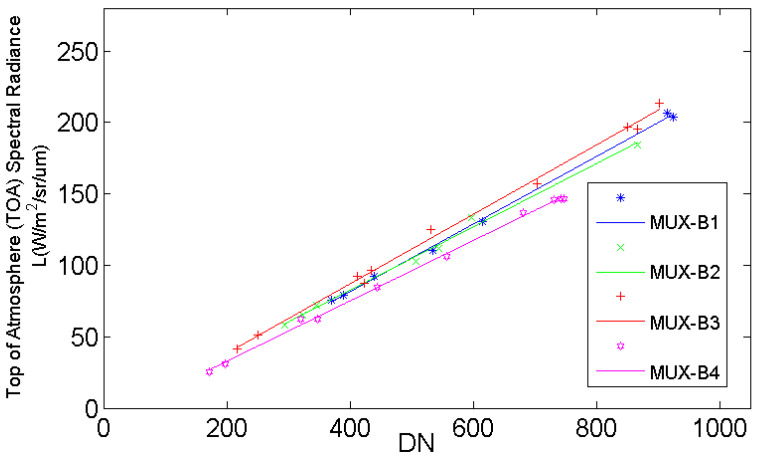
Calibration result of combining the permanent artificial targets and the radiometric calibration tarps (coefficient C).

The last coefficient (coefficient C, as shown in Figure 11) was determined by combining permanent artificial targets and radiometric calibration tarps, which means that there would be seven gray-scale targets and two color-scaled targets. All the calibration results show that linear correlations between DN and TOA radiances of ZY3-02 MUX are very high, with a correlation coefficient $R^{2}$ value of up to 0.99 for each band.

Table 5 shows the three different radiometric calibration coefficients and laboratory calibration coefficients.

### 4.2. Uncertainty Analysis of the Reflectance-Based Approach

Early work with Landsat-7 ETM+, Landsat-8 OLI, SPOT HRV, IKONOS, RapidEye, and KOMPSAT showed that the reflectance-based approach had an absolute radiometric uncertainty of ~5% [6,20,21,22,23]. The reflectance-based uncertainty table listed the major source of radiometric uncertainty of the reflectance-based approach. Detailed descriptions of the uncertainty were discussed as follows. There are several basic areas of uncertainty in the method: (1) Surface reflectance measurement, (2) Atmospheric characterization, and (3) Inherent accuracy of the MODTRAN 6.0 radiative transfer code.

The uncertainty in a single measurement of the surface reflectance with SVC HR1024 spectroradiometer is <2%. Based on the laboratory calibration tarp BRDF measurements, we can assign a 1.5% error accuracy for assuming a Lambertian calibration tarp. The view zenith angle of ZY3-02 MUX was less than 3 degrees, and the BRDF effects of the artificial calibration targets and calibration tarps were not considered in this study.

The second primary source of uncertainty in the reflectance-based approach is atmospheric characterization, which includes the aerosol optical depth measurement, total columnar water vapor, aerosol size distribution, and aerosol complex index of refraction. The AOD and CWV were retrieved by the AERONET data process center at the AOE Baotou site, and they led to the TOA radiance uncertainties of 0.5% for AOD and 0.4% for CWV. A standard rural aerosol type in the MODTRAN 6.0 radiative transfer code was used in this study. The two parameters of aerosol (the aerosol size distribution and the aerosol complex index of refraction) resulted in uncertainties of TOA radiance less than 2.0% for aerosol size distribution and 2.5% for aerosol complex index of refraction, respectively. The inherent accuracy of the MODTRAN 6.0 radiative transfer code is <1%.

All of the analyzed uncertainty sources above were assumed independent, and the overall uncertainty in the TOA radiance in this reflectance-based approach is 4.57%, less than 5%, as shown in Table 6.

### 4.3. Validation of Three Radiometric Calibration Coefficients

**Figure 12:**
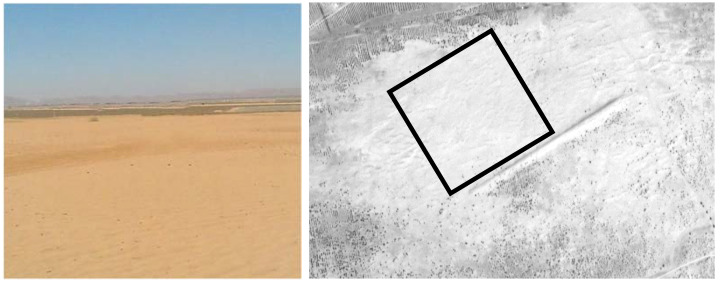
The desert area at Baotou calibration site.

For the automated radiometric calibration and validation of moderate and high-resolution satellite sensors, a desert area (300 m × 300 m) was established in October 2015. This is 1.8 km away from the permanent target region to the north-west and has been flattened. Figure 12 shows the desert area at Baotou site. The distance between the gray-scale permanent artificial targets and the desert area is approximately 3 km.

In order to validate the reliability of the three radiometric calibration results, the desert area (300 × 300 m^2^) was taken as a validation target. We obtain the surface reflectance of the desert and the atmospheric parameters (AOD and CWV). The TOA predicted radiance of the target $L_{predicted}$ can be calculated with the radiative transfer model MODTRAN.

(5) $$L_{predicted}=\frac{\rho_{\lambda }\cdot ESUN\cdot \cos\theta_{SZA}}{\pi \cdot d^{2}}$$

The average DN values of the desert area were extracted from ZY3-02 MUX imagery. The measured radiance $L_{measured}$ was radiometrically calibrated from DN with three different coefficients, and was compared with the TOA predicted radiance.

(6) $$L_{measured}=Gain_{i}\times DN+Bias_{i}$$

(7) $$\Delta L\%=\left|\frac{L_{measured}-L_{predicted}}{L_{predicted}}\right|\times 100\%$$

where $L_{measured}$ is the TOA measured radiance, and ΔL% is the relative difference between the TOA predicted radiance and the TOA measured radiance.

**Figure 13:**
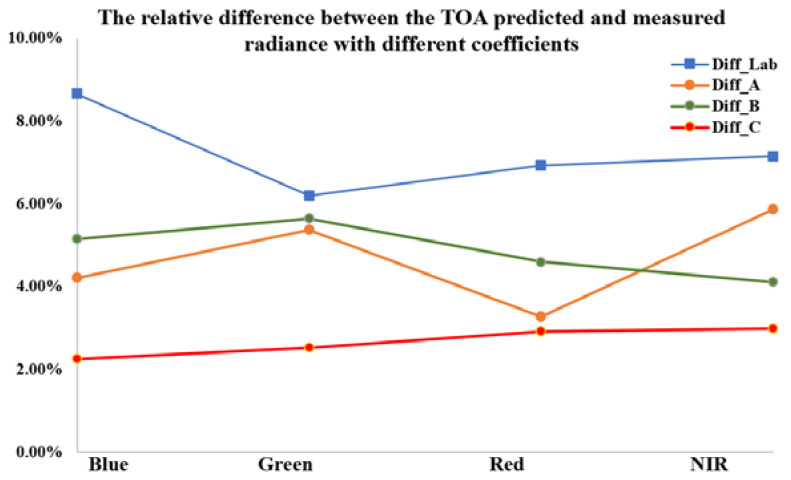
The relative difference between the TOA predicted and measured radiance with different coefficients.

With regard to the comparison between the measured radiance and TOA predicted radiance in Table 7, the relative difference between measured radiance C and TOA predicted radiance were lowest for the blue band (2.25%), followed by the green band (2.52%), red band (2.91%), and NIR band (2.98%), less than 3% in all bands (Figure 13). The calibration result of combining the permanent artificial targets and the radiometric calibration tarps (coefficient C) shows the best agreement between the measured radiance and TOA predicted radiance. Largest relative differences occurred in the laboratory radiometric calibration results, which indicated that the on-orbit radiometric calibration is a critical activity that must be regularly performed.

### 4.4. Cross-Validation with Landsat-8 OLI Using the Three Determined Radiometric Calibration Coefficients

**Figure 14:**
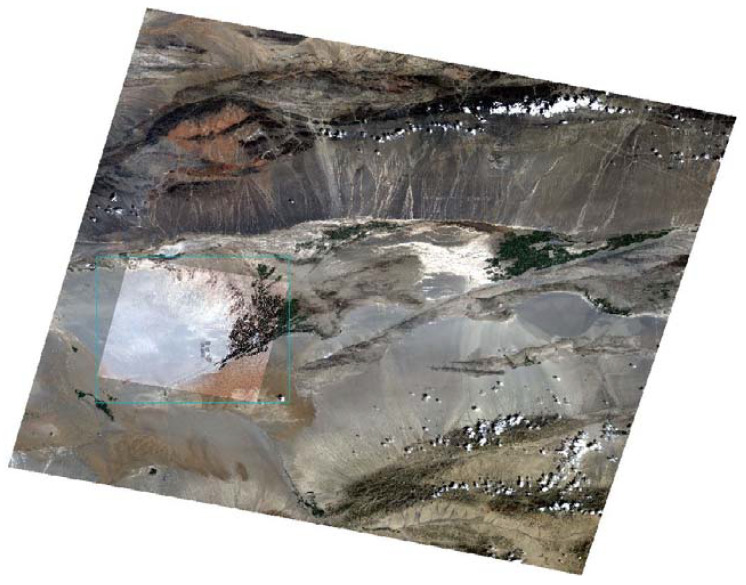
The Dunhuang test site in Landsat-8 OLI and ZY3-02 MUX imagery.

To further validate the reliability of the abovementioned three radiometric calibration results, the calibrated ZY3-02 MUX was compared with Landsat-8 Operational Land Imager (OLI), which is considered as a reference sensor of ZY3-02. The radiometric accuracy of Landsat-8 OLI is typically within 3% for all solar-reflective bands [24]. The Dunhuang test site was carefully selected as reference standard test site for cross-validation of ZY3-02 MUX with Landsat-8 OLI. The Dunhuang test site was China’s national radiometric calibration site, which had been used to calibrate the Chinese satellites such as Feng-Yun series of satellites and the Gao-Fen series of satellites [25]. Figure 14 shows the Dunhuang test site in Landsat-8 OLI and ZY3-02 MUX imagery.

**Figure 15:**
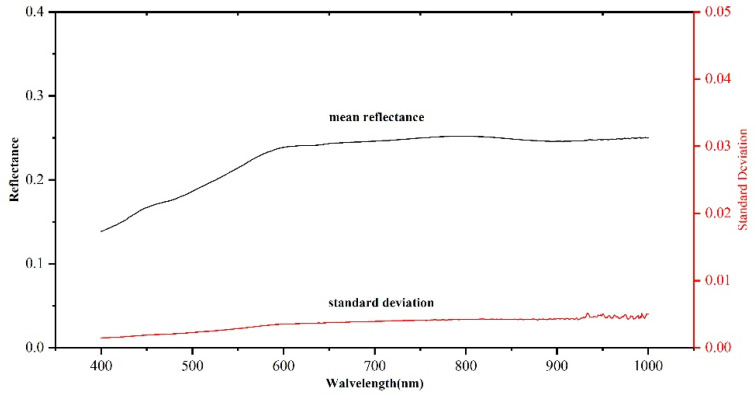
Surface reflectance and standard deviation of Gobi in Dunhuang test site.

The Dunhuang test site was used for cross-validation based on the stability and homogeneity of the Gobi Desert (Figure 15).

**Figure 16:**
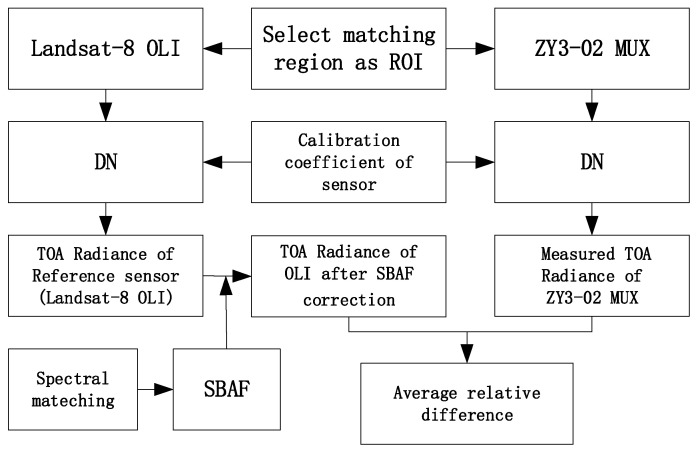
Flow chart of cross-validation with Landsat-8 OLI.

Figure 16 shows the flow chart of cross-validation with Landsat-8 OLI. The corresponding subset areas with 900 × 900 m from ZY3-02 and Landsat-8 were selected to acquire the homogenous areas within the Dunhuang Gobi site. In the case of ZY3-02 MUX, a total of 150 × 150 pixels were used, while Landsat-8 OLI was 30 × 30 pixels. Table 8 shows geometric conditions of ZY3-02 and Landsat-8 OLI for cross-calibration.

The TOA radiance of the ZY3-02 sensor is calculated by Formula (8).

(8) $$L_{measured}^{MUX}=Gain\times DN+Bias$$

where $L_{measured}^{MUX}$ is the TOA radiance at ZY3-02 MUX band, expressed in units of $W\cdot m^{-2}\cdot sr^{-1}\cdot \mu m^{-1}$. DN is the digital number from the reference satellite imagery. Gain and Bias are the official calibration coefficients in units of $W\cdot m^{-2}\cdot sr^{-1}\cdot \mu m^{-1}$.

As the relative spectral responses (RSR) of each band from “reference sensor” Landsat-8 OLI and “sensor to calibrate” ZY3-02 MUX were strictly different, the TOA radiance of Landsat-8 OLI imagery needed to be transferred to ZY3-02 sensor imagery with a spectral band adjustment factor (SBAF).

The differences in the RSR between different satellite sensor were compensated by SBAF, as in Formula (9).

(9) SBAFMUX/OLIRadiance=∫LMUX(λ)⋅RSRMUX(λ)dλ∫RSRMUX(λ)dλ∫LOLI(λ)⋅RSROLI(λ)dλ∫RSROLI(λ)dλ

The TOA radiances of the Landsat-8 OLI imagery were transferred to ZY3-02 sensor imagery by the formula which follows, in which $L_{OLI}^{SBAF}$ is the TOA radiance from “reference sensor” Landsat-8 OLI after SBAF correction.

(10) $$L_{OLI}^{SBAF}=L_{OLI}\times SBAF_{MUX/OLI}^{Radiance}$$

The average relative difference (ARD) between the TOA radiance from Landsat-8 OLI after SBAF correction $L_{OLI}^{SBAF}$ and the TOA radiance of the ZY3-02 MUX $L_{measured}^{MUX}$ is:

(11) $$ARD=mean(\frac{L_{OLI}^{SBAF}-L_{measured}^{MUX}}{L_{OLI}^{SBAF}})\times 100\%$$

**Figure 17:**
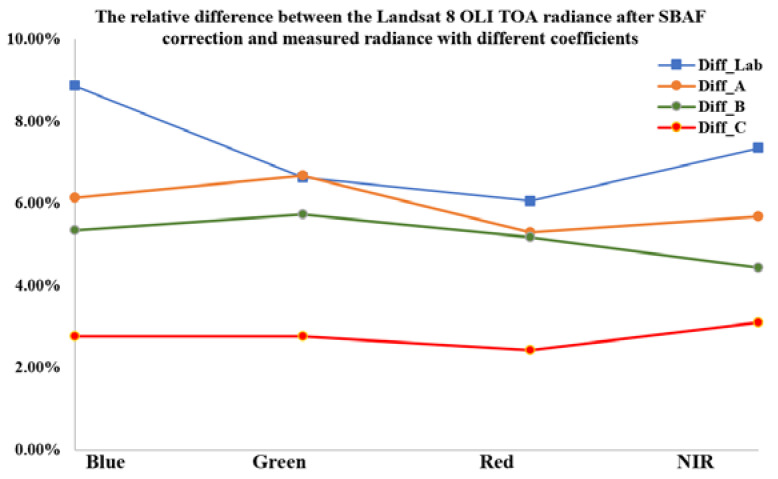
The relative difference between the Landsat-8 OLI TOA radiance after SBAF correction and the measured radiance with different coefficients.

Finally, the calibrated ZY3-02 MUX with coefficient C shows the best cross-validation results, and the average relative difference between ZY3-02 MUX Measured TOA Radiance and Landsat-8 OLI TOA radiance after SBAF correction is less than 3.5% in all bands of ZY3-02 MUX (Table 9, Figure 17). The Dunhuang Gobi cross-validation results also show good agreement with the Baotou desert area validation results. The results show that radiometric calibration coefficient C of combining the permanent artificial targets and the radiometric calibration tarps is best.

## 5. Conclusions

In this study, the methods and results of a reflectance-based vicarious calibration campaign were described. The relationship between the DN and TOA radiance exploiting radiometric calibration coefficients was obtained via an absolute radiometric calibration campaign at the RadCalNet Baotou site. We obtained three radiometrically calibrated coefficients of ZY3-02 MUX using different targets and tarps. The calibration results show that linear correlations between DN and TOA radiances of ZY3-02 MUX are very high, with a correlation coefficient $R^{2}$ value of up to 0.99 for each band. A detailed discussion on the uncertainty analysis of the radiometric calibration is presented in this paper, and the overall uncertainty of this reflectance-based approach is 4.57%.

The aim of our study was to obtain a reliable and high-accuracy radiometric calibration coefficient for ZY3-02 MUX. In order to consolidate the ZY3-02 MUX absolute radiometric calibration, the three calibration coefficients were validated by the synchronous measurement of surface reflectance and atmospheric characterization in the RadCalNet Baotou desert site, and also cross-validated with Landsat-8 OLI in the Dunhuang Gobi site. A detailed discussion on the validation analysis of the comparison results between the different radiometric calibration coefficients is presented in this paper. The reasonably good agreement of the radiometrically calibrated coefficients of the ZY3-02 MUX is encouraging, which shows that the percentage difference in coefficient C was within 4% in the MUX bands. The radiometric coefficient C was determined by combining permanent artificial targets and radiometric calibration tarps, which means that there would be seven gray-scale targets and two color-scaled targets. It should be noted that absolute radiometric calibration incorporating the permanent gray-scale target and calibration tarps significantly enhances the accuracy and quality. The results also indicate that radiometric characteristics of ZY3-02 are reliable and high-accuracy quantitative applications. The radiometric calibration coefficients would be useful for users when utilizing ZY3-02 MUX imagery.

## Figures and Tables

**Table 1:** The technical specification of ZY3-02 Satellite.

ZY3-02 Satellite	Technical Specification
Launch Schedule	May 2016
Orbit	Altitude: 506 km
	Type: sun-synchronous,
	Equator Crossing time: 10:30 AM
	Cycle: 97 min
Mission duration	5 Years
Image Band of Remote Sensor	Panchromatic and multi-spectral (Four-band: blue, green, red, NIR)
Spatial resolution	Panchromatic: nadir-view: 2.1 m (GSD)
	forward-view (+22°): 2.5 m (GSD)
	backward-view (−22°): 2.5 m (GSD)
	Multi-spectral: nadir-view: 5.8 m (GSD)
Dynamic Range	10 bits/pixel; time-delayed integral imaging
Swath width	51 km
Attitude determination and control	Three-axis stabilization
	Sensors: star sensor, solid inertia reference: GPS
Pointing Accuracy	0.1°
Side-sway Ability	Steer up to ±32°
Cycle	Repeat cycle time: 59 days
	Revisit time: 5 days

**Table 2:** The spectral responses properties of ZY3-02 MUX.

Band	Description	Spectral Range (nm)	Specified Spectral Range at 50% Transmittance Points(nm)	Center Wavelength (nm)	Bandwidth (nm)
1	Blue	450–520	462.3–512.2	490	70
2	Green	520–590	515.7–587.1	563	70
3	Red	630–690	625.8–693.0	676	60
4	NIR	770–890	763.9–885.4	807	120

**Table 3:** Synchronous measurement of atmospheric parameters.

Atmospheric Parameters	Synchronous Measurement
AOD @ 550 nm	0.1276
CWV	0.8763 g/cm^2^

**Table 4:** Geometric conditions of ZY3-02 for radiometric calibration.

Site	Date	Overpass Time (UTC)	Solar Zenith	Solar Azimuth	Viewing Zenith	Viewing Azimuth
Baotou	20 July 2016	03:45:08	34.687	140.411	1.71	47.459

**Table 5:** Different radiometric calibration coefficients (in units of $W\cdot m^{-2}\cdot sr^{-1}\cdot \mu m^{-1}$).

Coefficients	A		B		C		Laboratory	
**Band**	*Gain*	*Bias*	*Gain*	*Bias*	*Gain*	*Bias*	*Gain*	*Bias*
MUX-B1	0.2495	−18.36	0.2342	−17.07	0.2291	−11.62	0.2004	−0.1794
MUX-B2	0.2282	−10.55	0.2333	−13.60	0.2213	−6.241	0.2031	−0.3262
MUX-B3	0.2542	−15.48	0.2454	−12.95	0.2432	−10.29	0.2205	−0.1812
MUX-B4	0.2466	−23.33	0.2261	−16.40	0.2110	−9.552	0.2044	−0.4489

**Table 6:** Sources of uncertainties in TOA radiance in the reflectance-based approach.

Source of Uncertainty	Accuracy %	TOA Radiance Uncertainty %
Surface reflectance measurement	2%	2.0%
Lambertian assumption of targets	1.5%	1.5%
Aerosol optical depth		0.5%
Total columnar water vapor		0.4%
Aerosol size distribution		2.0%
Aerosol complex index of refraction		2.5%
MODTRAN 6.0 Radiative transfer	2.0%	2.0%
Overall Uncertainty		4.57%

**Table 7:** The relative difference between the TOA predicted and measured radiance (in units of $W\cdot m^{-2}\cdot sr^{-1}\cdot \mu m^{-1}$).

Band		Blue	Green	Red	NIR
TOA Predicted Radiance by MODTRAN 6.0 $L_{predicted}$		97.233	106.576	113.235	96.328
ZY3-02 MUX measured radiance $L_{measured}$	Laboratory Coefficients	105.642	113.173	121.068	89.449
	Coefficients A	101.327	112.289	116.940	90.686
	Coefficients B	102.243	112.587	118.436	92.375
	Coefficients C	99.419	109.267	116.530	93.456
Relative difference ΔL%	Diff_Lab	8.65%	6.19%	6.92%	7.14%
	Diff_A	4.21%	5.36%	3.27%	5.86%
	Diff_B	5.15%	5.64%	4.59%	4.10%
	Diff_C	2.25%	2.52%	2.91%	2.98%

**Table 8:** Geometric conditions of ZY3-02 and Landsat-8 OLI for cross-calibration.

Sensor	Date	Overpass Time (UTC)	Solar Zenith	Solar Azimuth	Viewing Zenith	Viewing Azimuth	AOD@ 550 nm	Cloud Condition
**ZY3-02** **MUX**	22 July 2016	04:31:48	24.966	137.774	0.899	186.249	0.0892	no cloud
**Landsat-8 OLI**	25 July 2016	04:20:20	27.759	132.077	0	51.462	0.1022	no cloud

**Table 9:** Comparison of results between ZY3-02 MUX Measured TOA Radiance using different calibration coefficients and Landsat-8 OLI TOA Radiance after SBAF correction.

Calibration Coefficients	Average Relative Difference (%)			
	Blue	Green	Red	NIR
**Laboratory** **Coefficients**	8.86%	6.63%	6.06%	7.35%
**Coefficients A**	6.14%	6.67%	5.30%	5.68%
**Coefficients B**	5.34%	5.73%	5.17%	4.43%
**Coefficients C**	2.76%	2.77%	2.43%	3.11%

## Data Availability

Not applicable.
